# Critical Analysis of the Current Medical Image-Based Processing Techniques for Automatic Disease Evaluation: Systematic Literature Review

**DOI:** 10.3390/s22187065

**Published:** 2022-09-18

**Authors:** Baidaa Mutasher Rashed, Nirvana Popescu

**Affiliations:** Computer Science Department, University Politehnica of Bucharest, 060042 Bucharest, Romania

**Keywords:** medical image analysis, machine learning, deep learning, diagnosis system

## Abstract

Medical image processing and analysis techniques play a significant role in diagnosing diseases. Thus, during the last decade, several noteworthy improvements in medical diagnostics have been made based on medical image processing techniques. In this article, we reviewed articles published in the most important journals and conferences that used or proposed medical image analysis techniques to diagnose diseases. Starting from four scientific databases, we applied the PRISMA technique to efficiently process and refine articles until we obtained forty research articles published in the last five years (2017–2021) aimed at answering our research questions. The medical image processing and analysis approaches were identified, examined, and discussed, including preprocessing, segmentation, feature extraction, classification, evaluation metrics, and diagnosis techniques. This article also sheds light on machine learning and deep learning approaches. We also focused on the most important medical image processing techniques used in these articles to establish the best methodologies for future approaches, discussing the most efficient ones and proposing in this way a comprehensive reference source of methods of medical image processing and analysis that can be very useful in future medical diagnosis systems.

## 1. Introduction

Classification methods have increased in importance and now play a significant role in image processing. Their importance stems from their applications in various fields, particularly in medicine. Given the importance of classification in medicine, new and sophisticated classification tools and methods are needed to diagnose and classify medical images efficiently [1]. Several classification algorithms encompass hundreds of different classification issues, and no single classification method can successfully and efficiently address all classification problems. As a result, answering the question concerning which classification approach is best for a particular study is challenging. The fast growth in medical data and imagery in recent years has necessitated the employment of new methodologies depending on big data technology, artificial intelligence, and machine learning in health care, making it an important research area [2]. Given the importance of classification in the medical field, new approaches for rapidly identifying and evaluating medical images are required. As a result, this research aims to compare existing and conventional methods for medical image classification and, based on these findings, suggest a novel algorithm for medical image classification [3]. 

The field of medical image processing and analysis has contributed to substantial medical achievements. A correct diagnosis necessitates the precise identification of each disease by integrating methods and techniques that support more effective clinical diagnosis depending on images obtained by various imaging modalities that have been used increasingly widely and successfully to detect illnesses [4]. This study aims to describe the process of medical image analysis, identify the techniques used in the analysis, and give a comprehensive literature review on illness identification based on medical imaging across various diseases and diverse fields and applications in medical imaging. This study searched for works related to the topic of the systematic literature review (SLR) and provided information about the process applied *to* article selection. In the final step, we kept only the forty most relevant articles that answered our research questions related to medical image processing and analysis techniques for diagnosis. 

The study is organized as follows. Section 2 describes the methodology of research applied in the SLR. Section 3 provides a detailed explanation of medical imaging modalities and medical image analysis processes in the surveyed studies. Here, the image processing methods for disease diagnosis developed by the researcher are described. Various filtering and image improvement methods are discussed. Popular segmentation methods are presented, feature extraction methods are introduced, and the classification methods utilized for human disease diagnosis and evaluation metrics are discussed. Section 7 discusses the outcomes and the future work. 

## 2. Research Methodology

This section describes the protocol utilized to locate, collect, and assess the state-of-the-art techniques under study. It is divided into four phases: research questions, research strategy, article selection criteria, and research results.

### 2.1. Research Questions

The SLR (Systematic Literature Review) aims to address the research questions by finding all relevant research outcomes from previous studies. The research questions are divided into five sub-questions:

Q1: What are the modalities of medical imaging?

Q2: What is the task of medical image processing and analysis?

Q3: Which medical image processing methods are most used in diagnostic systems?

Q4: What diagnostic techniques have been adopted and developed?

Q5: Is the system that has been adopted or designed capable of producing good results?

We searched various databases such as Elsevier, IEEE Explorer, Springer, and Google Scholar. We included the relevant studies that mainly focus on two or more questions based on our research questions.

### 2.2. Research Strategy

**Figure 1:**
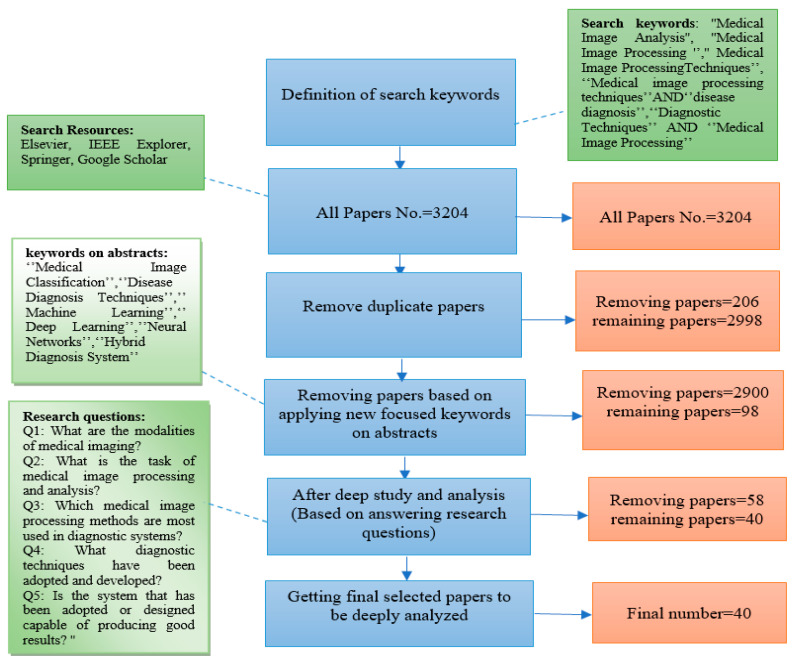
Flowchart of the process for article selection.

Our systematic literature review collected many studies related to our search topic over the last five years, between 2017 and 2021, from the following databases: Elsevier, IEEE Xplorer, Springer, and Google Scholar. We used combinations of keywords and terms, including “Medical Image Analysis”, “Medical Image Processing”, “Medical Image Processing Techniques”, “Medical Image Processing Techniques” AND “Disease Diagnosis”, and “Diagnostic Techniques” AND “Medical Image Processing”, and we obtained about 3204 articles meeting our searched keywords. After removing 206 duplicate articles, the remaining set consisted of 2998 articles. After examining these studies by their titles and abstracts, a new set of keywords were applied, including “Diseases Classification”, “Machine Learning”, “Deep Learning”, “Neural Networks”, and “Hybrid Diagnosis System”, and 2900 articles were excluded. Of the 98 articles remaining, 58 were excluded after deep reading and based on the exclusion criteria focused on articles that used machine learning and deep learning methods to create a hybrid diagnostic system based on the merging of two methods or the modification or improvement of a common method and its development for proposing possible areas for future research. These articles reached the fourth stage of our systematic exclusion technique and dealt with different topics and methods related to machine learning (ML) methods found in [5,6,7,8,9,10,11,12,13,14,15,16,17,18,19,20,21,22,23,24,25,26,27,28,29,30,31,32], deep learning (DL) strategies found in [33,34,35,36,37,38,39,40,41,42,43,44,45,46,47,48,49,50,51,52,53,54], and convolutional neural network (CNN) approaches found in [55,56,57,58,59,60,61,62]. Finally, 40 studies fulfilling our research criteria were obtained to be deeply analyzed. In this way, the most suitable articles were selected based on the PRISMA (Preferred Reporting Items for Systematic Reviews and Meta-Analyses) technique, as shown in Figure 1. By applying the PRISMA technique, which is appropriate for any systematic literature review, we kept only the most relevant articles from large databases. In the final step, as shown in Figure 1, the last article set was not only a result of the automatic selection based on keyword combinations but also represented the answers to our research questions that are discussed in Section 2.3. 

### 2.3. Criteria for Article Selection

The following criteria were determined to choose articles:

1. Articles using the most up-to-date techniques for analyzing medical images.
2. Articles that were written in the English language.
3. Articles published in the last five years (2017–2021).
4. Studies that were presented at a peer-reviewed conferences or journals.

Following the definition of the inclusion criteria, the following exclusion criteria were determined:

1. Duplicate references from the various electronic archives that were searched.
2. Articles with a page count of less than four.
3. Articles that fail to respond to any of the research questions.
4. Articles that were written in a language other than English
5. Articles that did not address the study’s goals.

### 2.4. Research Results

After searching the scientific databases and identifying the research results, the most relevant articles related to the research aims were found, which included articles that explored different medical image processing techniques and focused on the techniques of diagnosing diseases. The selected articles were carefully read, and the extracted results were analyzed and assessed to summarize the existing research, identify the most useful techniques, and propose possible areas for future research.

## 3. Results of Systematic Review

This section is divided into two parts: the first deals with medical imaging modalities, and the second deals with the analysis of medical images. Each revolves around the main objectives of the systematic review.

### 3.1. Medical Imaging Modalities

**Figure 2:**
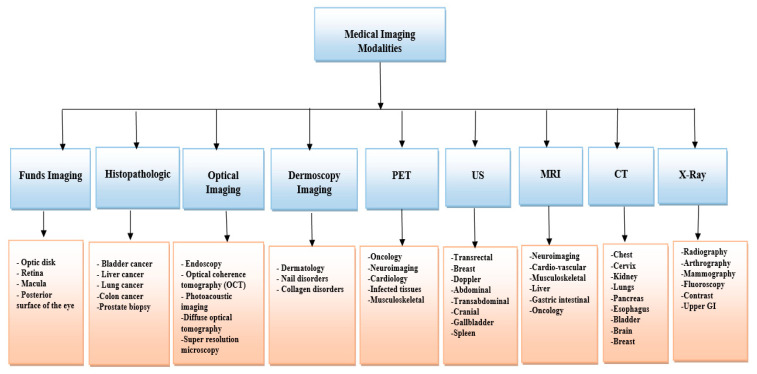
Classification of medical imaging modalities.

Medical images play a critical role in assisting health care workers in reaching patients for diagnosis and treatment. Medical image processing is a set of procedures for extracting clinically useful data from various imaging modalities for diagnosis [63]. Numerous medical imaging modalities include ionizing radiation, magnetic resonance, nuclear medicine, optical methods, and ultrasound as the media. Each modular media has unique characteristics and responses to the human body’s structure [64]. These modalities serve various purposes, such as obtaining images inside the human body or image samples of parts that cannot be seen with the naked eye [65]. The classification of medical imaging modalities and the main types of imaging methods addressed in the surveyed studies are illustrated in Figure 2.

The distribution of the forty chosen studies that used different modalities is illustrated in Table 1; this table shows the detailed distribution of publication references, imaging modality, type of disease, and medical databases.

### 3.2. Medical Image Analysis

This section is divided into subsections. These subsections introduce the major medical image analysis methods used in the studies reviewed, including image preprocessing, image segmentation, feature extraction to classification, and evaluation metrics.

#### 3.2.1. Medical Image Preprocessing

Image processing is a method for enhancing the quality of an image after eliminating irrelevant image data. Medical images include many irrelevant and unwanted segments. To remove these segments in an image, some preprocessing methods are required. Image preprocessing aims to improve the quality of the images contained in the dataset, which improves the results of segmentation and feature extraction methods [106]. This section presents the preprocessing methods of the studies surveyed. One of the most important techniques to improve medical images and remove noise is filtering. One of the most important filters used is the median filter [69,73,87,91,92,95,99], with its major benefit being to preserve the edges and remove noise. In [71], the authors used the synthetic minority over-sampling technique to generate synthetic samples from minor classes rather than simply replicating them, and they used the changing perspective of images technique to expand the dataset. To generate new images, computer vision techniques such as gray scaling, blurring, enhancing contrast, changing the color channel, sharpening, minimizing noise, and smoothing were used. In [73], the authors described in their study how to utilize pixel-wise interpolation and the modified quadratic transform-based Radon transform algorithm to improve the contrast of the images. In [73,85,100], the authors applied the contrast limited adaptive histogram equalization (CLAHE) approach and morphological operations to remove noises and improve the images. In [76,81], the authors used a fast adaptive median filter to eliminate the noise from medical images and maintain accurate information. In [77], binarization and thinning techniques were applied to the selected images to produce better images. In [80,88,89], the authors improved the noise removal process for medical images to obtain clear images using a Weiner filter for contrast adjustment. In [84], the authors developed two methods to convert the input color images to grayscale, denoise, enhance contrast, and normalize the histogram. The first method utilized only a green channel, while the second method depended on calculating the grayscale by taking an average of red, green, and blue channels computed using certain weights.

In [86,96,102], the authors addressed the normalization method to enhance the visual quality of images. In [87] the authors used noise removal techniques such as max-min filter, midpoint filter, quantum noise filter, alpha-trimmed mean filter, impulse noise filter, and wavelet thresholding methods for noise removal from images to smoothened them to obtain better images. In [91], the authors employed linear and non-linear image processing filters such as mean, Gaussian, Log, and fuzzy filters to eliminate noise from images. In [92], the authors presented the BBHE approach (i.e., Brightness Preserving Bi- Histogram Equalization), a method that decomposes the original image into two sub-images to overcome the drawback of histogram equalization. This is accomplished using a gray-level and histogram equalization approach for each sub-image. The Gradwrap algorithm, addressed in [93], improves the image appearance by removing image distortions. The B1 non-uniformity algorithm corrects the image color and intensity information, and then the N3 bias field correlation is applied after the Gradwrap and B1 non-uniformity algorithms to correct intensity distortion. In [94], the authors used unsharp masking, a popular image sharpening method, to improve the contrast of histopathological images. In [97] the Wang–Mendel algorithm was employed to remove noise from the images; this algorithm is one of the most effective approaches for eliminating noise from medical images due to its high speed.

In [101], the authors analyzed fundus images for image scaling; R, G, and B channel selection; and the preprocessed green channel components were analyzed by the 2D-VMD technique, which has a non-stationary, non-recursive, and completely adaptive decomposition technique for signal image analysis. In [103], the authors used image normalization and data over-sampling techniques with data augmentation to enlarge the dataset so that it could be used for deep learning tasks. Data augmentation approaches use a variety of operations to images, including scaling, geometric deformation, noise addition, alterations to the lighting, and image flipping. The authors employed the straightforward and efficient semi-supervised learning technique of pseudo-label to improve the performance of deep neural network models.

#### 3.2.2. Segmentation Techniques

Image segmentation is responsible for recognizing and outlining items of interest in input images. Automatic medical image segmentation aids in the diagnosis of diseases and the identification of pathogens. Image segmentation methods can be divided into machine learning methods such as supervised and unsupervised machine learning and classical segmentation methods such as threshold-based, edge-based, and region-based methods [107]. This section presents the segmentation methods used by the studies surveyed. In [69,73,74,76,77,81], the authors used thresholding. In this method, the image is partitioned into its foreground and background depending on the threshold value. In [69,96], the authors applied the contour technique; active contour is an active segmentation model that separates the pixels of interest from an image using energy forces and limits. In [79,91], the authors suggested using watershed segmentation techniques; this approach is used to separate different objects. The study [82,91] employed Otsu’s threshold approach to extract the object; this approach finds an optimum threshold automatically. Because of its simple calculation, Otsu’s is the most successful technique for image thresholding. The authors in [84] used the Gaussian matched filter approach with binarization realized with local entropy thresholding. This method allowed them to acquire more exact results than using only one binarization threshold. Additionally, the authors employed the crossing number algorithm or minutiae detection to extract minutiae and then automatically count their number.

The study [95] adopted a color space-based method for mammography image segmentation, followed by mathematical morphology. Post-processing mathematical morphology improved performance, including filling, closure, and other operations.

#### 3.2.3. Feature Extraction Techniques

Feature extraction is the process of extracting meaningful data from raw data. It is crucial in image processing as it enhances the image’s quality by reducing the dimensionality of the image, extracting the unique features, and transforming the input data into a set of features that are used for classification purposes. There are various features such as color, texture, and shape, and each type has several methods to extract features from medical images [108]. The distribution of the chosen studies that used different feature extraction and reduction methods is illustrated in Table 2; this table comprises the detailed distribution of publication references, the type of features, and the method used.

#### 3.2.4. Classification Techniques

Image classification is a difficult task in image analysis. The primary purpose of medical image classification is to accurately establish which parts of the human body are infected with the disease [109]. This study reviews the chosen studies that used the newest classification techniques for medical image classification. The study [67] proposed a new classifier depending on KPCA and SVM. The support vector machine with kernel principal component analysis (SVM-KPCA) approach was designed to classify the images, and the proposed method achieved effective results, which gave 100% accuracy, 100% sensitivity, and 100% specificity. In the study [68], the authors suggested a hybrid approach consisting of a radial basis function neural network (RBFNN) to classify brain MRI images, the accuracy of this model varied from 80% to 90%.

The study [70] developed a neural-based detection system by employing skin imaging for two different skin disorders. The ANN was trained using the non-dominated sorting genetic algorithm-II, a well-known multi-objective optimization technique (NN-NSGA-II). The proposed model was compared to two well-known metaheuristic-based classifiers, NN-PSO (ANN trained with PSO) and NN-CS (ANN trained with Cuckoo Search). The proposed bag-of-features enabled the N-NSGA-II model and obtained 90.56% accuracy, 88.26% precision, 93.64% recall, and 90.87% F-measure with the experimental data, indicating its superiority over other models. The study [71] applied multiple artificial intelligence (AI) techniques, such as the convolutional neural network and support vector machine, which were combined with image processing tools to construct a superior structure; the accuracy attained after training with CNN alone was approximately 91%, which was raised to approximately 95.3% when combined with the SVM. In [79], the authors developed a new SVM-FA (support vector machine optimized with firefly technique) classifier for diagnosing lung cancer in CT images where the SVM classifier, optimized with the firefly technique, was applied to the preprocessed data. A comparative analysis was conducted between the proposed work, traditional work, and the SVM classifier to assess the competence level of the proposed SVM-FA technique. The suggested work was successful and efficient, achieving an accuracy of 96 % and specificity of 83.3%.

The study [86] applied a deep neural network (DNN) with the rectified Adam optimizer to detect Alzheimer’s disease in MRI images. The experimental outcomes showed that DNN with the rectified Adam optimizer outperformed the existing work, landmark-based features with the SVM classifier, with a 16% classification accuracy. In a study [92], the authors addressed a combination of MLC (maximum likelihood classifier) and SVM (support vector machine) classifiers for the classification and diagnosis of DR (diabetic retinopathy) disease in the fundus images. The researchers utilized these classifiers to raise the performance level, and the suggested approach demonstrated high accuracy (98.60%), sensitivity (99%), and specificity (99%). In [95], the authors introduced an individual classifier called: feed-forward ANN (FF-ANN) and two hybrid classifiers, namely: random subspace with random forest (RSwithRF) and random subspace with Bayesian network (RSwithBN), for the classification of MRI brain images. The proposed system showed that ANN and hybrid classification approaches are the most appropriate for classification because of their high accuracy rates and achieved an accurate classification of 95.83%, 97.14%, and 95.71%, respectively.

In [100], the authors used various techniques, such as machine learning and different deep learning models, to predict various ophthalmic diseases; the study showed that the efficiency of each strategy varied depending on the input dataset and based on the many symptoms of each disease.

#### 3.2.5. Metric Evaluation

Accuracy, specificity, and sensitivity are the most prevalent metrics utilized in most disease diagnosis applications for humans. The number of true positive (TP), true negative (TN), false positive (FP), and false-negative (FN) samples are used to calculate these measures [110]. The sensitivity determines how many positive samples were identified (TP), whereas the specificity determines how many negative samples were identified (TN). The ratio of correctly recognized samples to the total number of samples is used to calculate classification accuracy [110]. Other metrics such as precision, recall, F1 score, and AUC must be used in conjunction with these metrics [111]. Table 3 illustrates the number of times each performance metric was utilized in each study.

## 4. Machine Learning Techniques

This study classified machine learning (ML) segmentation and classification techniques as supervised and unsupervised machine learning. Supervised learning algorithms generate mathematical models using a set of labeled data (images) which are utilized for training, examples of supervised machine learning algorithms are K-nearest neighbors (K-NN), support vector machines (SVM), decision trees (DT), linear regression, logistic regression, random forest (RF), artificial neural networks (ANN), gradient boosting, and naïve Bayes models. Unsupervised learning algorithms create mathematical models based on a set of data that only contains inputs and no required output labels. The algorithms identify patterns in the data and classify them. Examples of unsupervised machine learning algorithms are K-mean clustering, hierarchical clustering, Apriori algorithm, principal component analysis (PCA), and fuzzy C-means (FCM) [112]. 

In the surveyed studies, several techniques of machine learning (ML) in segmentation and classification were deployed. The study [85] compared the IPFCM (intuitionist possibilistic fuzzy C-mean) method with the hybridization of the negative function of the intuitionistic and the negative function of the possibilistic for mammogram image segmentation with conventional segmentation methods such as the Otsu algorithm, FCM (fuzzy C-mean) clustering, IFCM (intuitionist fuzzy C-mean) clustering, and PFCM (possibilistic fuzzy C-mean). Moreover, they found that this proposed method was the best approach. In studies [88,91,98], the authors used the fuzzy clustering method. Fuzzy clustering is the most extensively used for image segmentation because of its benefits compared with traditional clustering methods, which includes: making regions more homogeneous, decreasing the number of erroneous spots, reducing noise sensitivity, and removing noisy regions [88]. In [92], the authors introduced the MSW-FCM technique (modified spatial weighted fuzzy C-means) for accurately segmenting blood vessels in retinal fundus images. 

The distribution of the chosen studies that used the most popular machine learning techniques to diagnose human body disease is illustrated in Table 4. This table comprises the detailed distribution of publication references, techniques used, classified tasks, and classification accuracy results.

## 5. Deep Learning

Deep learning (DL) is well-known for its performance in image segmentation and classification models [113]. Convolution neural networks (CNN) are the most extensively utilized deep learning approach in the articles reviewed. CNN is an important image processing approach that allows for accurately classifying aberrant and normal samples [114]. CNN employs a layered perceptron-driven architecture composed of fully connected networks in which every neuron in one layer is coupled to all neurons in the subsequent layers. The input images, an in-depth feature extractor, and a classifier are the three main components of a CNN [111]. There are three kinds of layers in CNN, each of which performs a different function: (1) convolutional, (2) pooling, and (3) fully connected. The convolutional layer extracts the characteristics of the structure. The fully connected layer then decides which class the current input belongs to, depending on the retrieved features. Then, the pooling layer is responsible for shrinking feature maps and network parameters. Transfer learning algorithms can increase CNN performance in the case of limited input data [115]. A CNN can be created from scratch using an existing pre-trained network without retraining or fine-tuning a pre-trained network on a target dataset [111].

According to this review, some research publications used distinct deep neural network architectures; Table 5 summarizes studies that used deep learning in disease diagnosis.

The study [66] suggested a new classification structure that depends on a combination of 2D CNN and recurrent neural networks (RNN) that learn the properties of 3D PET images by decomposing them into a series of 2D slices. The intra-slice characteristics are captured using hierarchical 2D CNNs, while the inter-slice features are extracted using the gated recurrent unit (GRU) of an RNN for final classification. The experimental outcomes showed that the suggested method has promising performance for Alzheimer’s disease (AD) diagnosis. The authors in [80] applied CNN-based algorithm on a chest X-ray dataset to detect pneumonia. Three approaches were examined, a linear support vector machine classifier with local rotation and orientation-free features, transfer learning on two CNN models: Visual Geometry Group, i.e., VGG16 and InceptionV3, and a capsule network training from scratch. Data augmentation is a data preprocessing technique applied to all three approaches, the outcomes revealed that data augmentation is an effective technique for all three algorithms to improve performance and efficiency. In [86], the authors proposed a successful approach for predicting the probability of brain cancers in MRI images using CNNs and the (Adam) optimizer algorithm. An adaptive moment estimation (Adam) optimizer has been introduced to expedite training the network and evaluate the model to attain maximum accuracy. The study [93] introduced a new deep learning-based CNN model created by the Bayesian optimization algorithm for classifying Alzheimer’s disease, mild cognitive impairment (MCI), and cognitively normal (CN) in MRI images. The proposed method gives extraordinary outcomes compared to the existing techniques. The study [97] compared a new optimized version of CNN and a new, improved metaheuristic, named the advanced thermal exchange optimizer for the detection of breast cancers with three different techniques including multilayer perceptron (MLP), multiple instances (MI), and transfer learning (TL), which were applied in the MIAS mammography database to demonstrate the superiority of proposed method. 

In a study [102], the authors suggested using the ML approach in the training process to create a prediction model by training the CNN algorithm in addition to using the “Adam” optimizer from the Python Keras optimization library that has an initial learning rate of 0.001. The evaluation was carried out after partitioning the data into 80% and 20% for training and evaluation, respectively, to compute the accuracy of classification and loss of model over a set number of 10 epochs, the proposed algorithm gave good results. In the study [103], the authors developed a transfer learning-based technique to determine the severity of diabetic retinopathy; the proposed model was a deep learning model that combined multiple pre-trained image classification CNN models with the global average pooling (GAP) technique. The accuracy of the model attained 82.4% quadratic weighted transfer learning kappa (QWK). In [104], the authors proposed a diagnosis system based on deep neural networks and image retrieval method. Transfer learning and hashing functions increased the CNN performance and image retrieval algorithms. The proposed system attained an accuracy of 97% for CNN and a content-based medical image retrieval (CBMIR) method. The study [105] compared the effectiveness of modern CNN models for the task of modality classification and reported the superiority of a deep learning-based method over classic feature engineering approaches based on multi-label learning algorithms. The experimental results demonstrated that deep learning is more efficient than traditional methods and produced better and more robust feature representations when compared to handcrafted feature extraction approaches. The findings showed that deep transfer learning techniques work well in the medical field, where data is scarce. The Google Inception-v3 model performed the best when it came to classifying medical picture modalities. Except for VGG-16 and Res-Net-50, the other models behaved similarly to Inception-v3.

Generally, the studies utilized accuracy, specificity, sensitivity, precision, recall, f1 score, and AUC as evaluation metrics. According to the conclusions of the studies, deep learning algorithms achieved good results in most of the evaluation metrics (as in Table 5).

## 6. Diseases Diagnosis System

**Figure 3:**
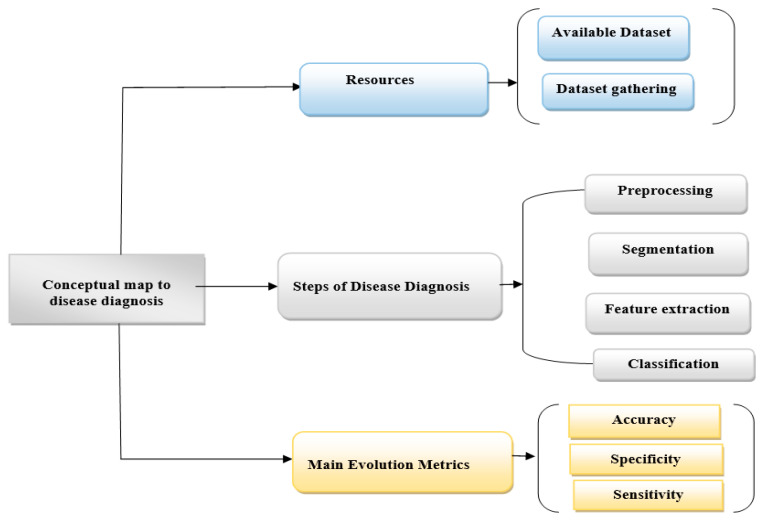
Conceptual map of disease diagnosis system.

Image processing has been extensively employed in various illness diagnosis procedures (human, animal, and plant), helping professionals select the appropriate treatment. In diagnosing human diseases, image processing techniques play an essential role. They can be used to identify disease signs (on the skin, for example) or in molecular research using microscope images that show the anatomy of the tissues [116]. The disease diagnosis systems comprise stages and methods for diagnosis. It starts from the first stage, which is the stage of collecting images from different sources, either through a database available online or collecting the images from different sources, i.e., images of patients available on the internet or from a specific hospital. Then the preprocessing image stage as different filtering methods are applied to improve the image, and then the segmentation stage follows by isolating the regions of interest and extracting the important features in the feature extraction stage. At the end of the process, the input image is classified and metrics for evaluating the effectiveness of disease diagnosis techniques are applied. The general system stages for the diagnosis of any disease in image processing are illustrated in Figure 3. This figure shows a conceptual map that explains concepts linked to disease diagnosis steps and a descriptive brief of the major evaluation metrics.

In this study, several disease diagnosis methods were studied that used different image treatment and classification strategies, which dealt with diagnosing human diseases, to examine the new and important methods that addressed in the studies.

## 7. Discussion and Future Directions

The growing interest in employing medical image processing approaches and AI techniques, such as ML and DL, may reduce the doctor’s workload and repetitive and monotonous procedures to diagnose and analyze patient data and images [117]. In this section, we discuss the state-of-the-art approaches and datasets used to diagnose human diseases through an answer to research questions and future directions.

### 7.1. Answer to Research Questions

The data were extracted from the studied articles as they were explained and clarified to answer the research questions.

“Q1: What are the modalities of medical imaging?” The methods of medical imaging were identified in the articles, where many modern imaging methods for diseases have been used, which help in diagnosing diseases in the early stages. The MRI imaging modality was used in fourteen articles in the diagnosis of brain diseases, lung diseases, cardiovascular diseases, and cardiac attacks; the CT imaging modality was used in eight articles in the diagnosis of lung diseases, liver tumors, and bone diseases. The X-Ray modality was utilized in seven articles to diagnose osteoarthritis disease, pneumonia disease, and breast cancer. Five articles used retina fundus images to diagnose eye illnesses for retina disease diagnosis. PET imaging was utilized in four articles to diagnose Alzheimer’s disease, and dermoscopy skin imaging was used to diagnose skin problems. 

“Q2: What is the task of medical image processing and analysis?“ In the analysis of the articles, it was identified that the task of the analysis of medical images and processing it to diagnose several diseases in different parts of the human body to assist the patient in detecting the disease and treating it in the early stage.

“Q3: Which medical image processing methods are most used in diagnostic systems?” Through the analysis of the articles, we discovered that by using filtering techniques, we may improve the initial image and get a more exact detection of the ROI borders. Noise can be reduced by smoothing with a low pass or median filtering, while edge sharpening and increased contrast can help to keep the ROIs’ borders. Color normalization, such as histogram equalization or specification, can boost contrast. A variety of transforms might be used to extract important features. Fourier and wavelet transform can be utilized to locate conditions in a domain other than the spatial one. Because the borders of the object or regions containing the crucial information for the diagnosis must be identified correctly, image segmentation is one of the most essential phases in a disease diagnosis procedure. The simplest but effective method is gray-level segmentation utilizing a single, multiple, or more advanced (Otsu) criteria. Several human illness diagnosis approaches have used active contour detection and modifications, such as a snake. Several clustering approaches were applied in the segmentation process, such as K-mean and fuzzy C-means, to separate the ROIs. The articles used many methods to extract the important features from objects. We found that the texture features were the most used in analyzing the articles. It was obvious from the analysis of the articles that the most popular classification method used in articles was a neural network with all types and SVM, where it was used alone or with another technique as a hybrid system for the classification of diseases. The rest of the classification techniques examined in this review such as K-means, K-NN, decision trees, naïve Bayes, random forest, logistic regression, and gradient boosting. All the classification or clustering methods can be exploited in the last stage of the diagnosis application to create a new diagnosis system to identify disease.

“Q4: What diagnostic techniques have been adopted and developed?” Analyzing the articles, we found that some of the articles adopted new hybrid methods for classification and diagnosis of diseases, such as using the SVM method with KPSA, SVM with FA, SVM with MLC, as well as using it with fuzzy. Moreover, the combination of random forest (RF) with random subspace (RS) and the networks CNN with RNN and with optimizers were used to create systems for disease diagnosis.

“Q5: Is the system that has been adopted or designed capable of producing good results?” The main classification methods discussed in the articles achieved efficient and good results in diagnosing diseases. The accuracy of SVM ranged between 88% and 100% in the several human disease diagnosis applications examined, naïve Bayes achieved accuracy between 78% and 94%, decision trees ranged between 82% and 99%, and random forest ranged between 80% and 99%. The different kinds of neural networks achieved accuracy between 73% and 97%, and the accuracy of CNN ranged between 86% and 99.3%. Finally, the accuracy of the K-nearest neighbor ranged between 73% and 95.5% in the referenced approaches.

### 7.2. Future Directions

It takes great effort to improve the performance of classification or diagnosis of diseases using multiple methods of medical images. In future work, we will try to present new research directions that can be further exploited in disease diagnosis through medical image processing techniques. We will compare the most common methods that are used in the diagnosis systems and select the most effective methods that introduce higher accuracy to the diagnosis for the medical image database that we use to build a new diagnostic system for diseases. The future research directions are discussed briefly as follows:

A. Study and analysis of the best and most common classification and diagnosis methods.

B. Experimenting with a set of medical data to compute the classification accuracy of the methods used.

C. Comparison of classification accuracy of these methods to identify the methods that are highly accurate.

D. Building a new diagnostic method by deriving a hybrid method, modifying a previous method, or combining previous methods.

The current study improved our knowledge of finding the best techniques that can be beneficial to our future research. We hope that this systematic literature review will also be useful to other researchers in their endeavor to improve disease diagnosis through medical image processing techniques. Due to our comparisons, the most common methods used in diagnosis systems have been discussed, making it easier to select effective methods that introduce higher accuracy to diagnosis methods using medical databases.

## 8. Conclusions

Medical imaging plays a critical role in inspecting and diagnosing human diseases. For diagnosis, many algorithms based on diverse methodologies have been created. As a result, disease detection has become an important topic in medical image processing and medical imaging research.

This review studied the articles on disease diagnosis published between 2017 and 2021. Overall, forty articles were analyzed from specialized academic repositories. The review focused on six factors: datasets used, various medical imaging modalities, image preprocessing techniques, image segmentation techniques, feature extraction techniques, classification approaches, and performance metrics, which were used to build and evaluate the disease diagnostic models. In addition, this systematic review highlighted a variety of AI techniques and presented a comprehensive study by exploring new diagnosis techniques, disease diagnosis issues, provided a variety of insightful information (such as the use of ML and DL), and an evaluation for each study. We aimed to address our study questions about effective diagnosis methodologies and discover the solutions proposed by many researchers in the diagnosis of diseases based on this. It was discovered that developing a new disease diagnosis method is quite important.

According to the findings of this SLR, researchers have adopted various methods to classify medical images associated with multiple disease diagnoses. These methods have shown promising results in terms of accuracy, cost, and detection speed. We found after analyzing the forty articles that the best preprocessing technique was the median filter, which was used in many studies, as has proven its ability to reduce noise and preserve the boundaries of the object. Regarding segmentation approaches, threshold techniques were the most used to extract the lesion from an image. Threshold-based approaches are the most often utilized among all the traditional methods, according to classic review publications, due to their applicability for numerous segmentation issues in medical images [118]. The methods for extracting features from medical images depend on the images used by analyzing the articles, we found that extracting texture features gave the best results. As for the applied classification methods, we found that the support vector machine method gave the best result in classification, as its accuracy in [67] reached 100% when it was used with KPC (support vector machine with kernel principal component analysis (SVM- KPCA)) approach. Thus, this method achieved the best accuracy and the best performance in diagnosis. Among the machine learning-based approaches whose associated works and analyses are presented, the supervised learning methods, notably the neural network-based methods, were the most widely employed, with different kinds of neural networks used to identify various diseases.

We also discovered that deep learning utilizing the CNN network has unique skills and advances in recognizing and classifying medical images, particularly those connected to breast, lung, and brain cancer. Other common classification approaches comprise fuzzy clustering, K-NN, K-means, decision trees, random forests, and other prominent classification algorithms. The most utilized measures were accuracy, sensitivity, and specificity.

This study aimed to propose future research directions by focusing on imaging modalities, techniques, and procedures used in the reviewed articles. Furthermore, this publication will aid in the development of new research that assesses and compares various medical image processing and analysis techniques. The findings provided in this SRL reveal that tremendous progress has been made in medical image processing over the last five years. In addition, the goal of this SRL was to undertake a detailed analysis of research achievements linked to the usage of medical image processing techniques applied to medical databases to know the current state-of-the-art techniques.

## Figures and Tables

**Table 1:** Distribution of studies for different medical imaging modalities.

Studies (Author (Year) [Ref])	Imaging Modality	Type of Disease	Medical Database
Danni Cheng et al. (2017) [66]	PET	Alzheimer’s disease	Alzheimer’s Disease Neuroimaging Initiative (ADNI) database (https://adni.loni.usc.edu/*)* (accessed on 18 June 2022). The dataset contained 339 brain images (93 AD, 146 MCI, 100 NC subjects).
Syrine Neffati et al. (2017) [67]	MRI	Brain disease	Harvard Medical School website, Open Access Series of Imaging Studies (OASIS) website. The dataset contained normal brains and seven types of pathological brains with a total of 226 images (38 normal brains and 188 pathological brains).
Varun Jain et al. (2017) [68]	MRI	Brain tumor	SICAS Medical Image Repository dataset contained 25 MRI brain images (20 benign, 5 malignant).
Anjukrishna et al. (2017) [69]	CT	Liver cancer tumor	Travancore scan center, Thiruvananthapuram (www.liveratlas.org) (accessed on 19 June 2022). The dataset contained 80 abdominal CT images, 20 of a normal liver and the rest images of various liver diseases.
Shouvik Chakraborty et al. (2017) [70]	Dermoscopy skin imaging	Skin cancers	International Skin Imaging Collaboration (ISIC) dataset contained images of two classes (skin angioma and basel cell carcinoma).
Soumya Sourav et al. (2017) [71]	Dermoscopy skin imaging	Dermatological diseases	Dermatology Online Atlas (www.dermis.net), http://homepages.inf.ed.ac.uk/rbf/DERMOFIT/ (accessed on 19 June 2022). The dataset contained 3000 images of four types (psoriasis, herpes, eczema, and melanoma).
Priyanka Lodha et al. (2018) [72]	MRI PET	Alzheimer’s disease	ADNI database (http://adni.loni.usc.edu) (accessed on 18 June 2022). The ADNI study was applied to people between the age of 55 and 90.
Keerthana T K et al. (2018) [73]	MRI	Brain tumor	Brain MRI medical image dataset which contained normal, benign, and malignant images.
Latika A. Thamke et al. (2018) [74]	CT	Lung diseases	CT scan image dataset collected from patients (age ranges from 35 to 75). The datasets contained 400 images (100 normal, 100 pleural effusion, 100 bronchitis, and 100 emphysema).
Abid Sarwar et al. (2018) [75]	Medical data (non-image)	Diabetes type-II	The authors prepared a rich database that included two classes (diabetic and non-diabetic) of 400 people from a large geographical area (age ranges from 5 to 75).
Pallavi. B et al. (2019) [76]	Dermoscopy imaging	Malignant melanoma skin cancer disease	The authors gathered specimen images of the sickly greeneries, then trained and stored them in the database. This database contained normal and abnormal images.
Neeraj Kumar et al. (2019) [77]	CT	Bone disease (osteoporosis)	The NCBI dataset associated with osteoporosis. The authenticated medical center Medpix NLM website. The database contained two classes with features (plane, modality, age, fracture, gender, weight, and history).
Rabi et al. (2019) [78]	Endoscopic images	Gastrointestinal (GI) diseases	The KVASIR dataset consisted of 4000 images containing 8 classes of GI diseases. Some of the supplied image classes feature a green image depicting the location and form of the endoscope within the intestine.
Sakshi Sharma et al. (2019) [79]	CT	Lung cancer disease	Database gathered from the IMBA web page contained normal and abnormal CT images of cancers for both males and females.
Smir S. Yadav et al. (2019) [80]	X-ray	Pneumonia disease	The dataset was based on previous literature. The dataset contained 5856 images (normal, bacteria, and viruses).
Sannasi Chakravarthy et al. (2019) [81]	CT	Lung cancer disease	Lung Image Database Consortium (LIDC). The dataset was composed of diagnostic and cancer screening thoracic CAT examinations with marked-up interpretations.
Aamir Bhat et al. (2019) [82]	X-ray	Osteoarthritis disease	The datasets were gathered from numerous hospitals. The dataset contained 126 knee joint X-ray images.
Mohammed Aledhari et al. (2019) [83]	X-ray chest radiographs	Pneumonia disease	National Institute of Health (NIH) dataset contained 1431 labeled X-ray images (normal and pneumonia).
Maciej Szymkowski et a l. (2020) [84]	Retina color images	Retina disease diagnosis	Medical University of Bialystok (MUB) Clinic Hospital, publicly available DRIVE STARE, Kaggle. The database contained 500 images (250 healthy samples and 250 pathological samples).
Shashank Awasthi et al. (2020) [85]	MRI	Alzheimer’s disease	The publicly available OASIS dataset contained MRI images of normal and AD patients.
Halebeedu Suresha et al. (2020) [86]	MRI	Alzheimer’s disease	The National Institute of Mental Health and Neurosciences (NIMHANS) dataset contained 800 images for 99 people (60 normal and 39 AD with age ranges from 55 to 87 years). The ADNI dataset contained 819 subjects (229 normal, 192 AD, and 398 MCI).
Chiranji Lal Chowdhary et al. (2020) [87]	Breast X-ray (mammogram)	Breast cancer disease	Mammography Image Analysis Society (MIAS) dataset contained 320 mammogram images (51 malignant, 63 benign, and 206 normal).
Fateme Gholami et al. (2020) [88]	MRI	Brain tumors	MRI image dataset gathered by the authors. The number of samples considered for evaluation in this article was 30 gray images.
Jaspreet Kaur et al. (2020) [89]	CT PET	Detecting cancer	PET Center, Postgraduate Institute of Medical Education and Research (PGIMER), Chandigarh, India. The dataset contained 200 medical images.
Begüm Erkal et al. (2020) [90]	Medical data (non-image)	Brain cancer	Broad Institute (http://portals.broadinstitute.org/cgi-bin/cancer/datasets) (accessed on 20 June 2022). The dataset contained 42 samples (7129 features and 5 classes).
G U Santosh Kumar et al. (2021) [91]	MRI	Cardiovascular diseases, cardiac attack	The dataset of patients from the York university contained the cardiac MRI DICOM images of patients suffering from various cardiovascular diseases.
Nasr Gharaibeh et al. (2021) [92]	Retinal fundus images	Diabetic retinopathy (DR)	The Image-Ret database included two sub-databases (i.e., DIARETDB0 and DIARETDB1).
Laiba Zubair et al. (2021) [93]	MRI	Alzheimer’s disease	The ADNI dataset contained 145 MRI images (39 AD, 45 CN, and 68 MCI).
Mehedi Masud et al. (2021) [94]	Histopathological image	Lung cancer and colon cancer	The LC25000 dataset contained 25,000 color images of five types of lung and colon tissues (colon adenocarcinoma, benign colonic tissue, lung adenocarcinoma, benign lung tissue, and lung squamous cell carcinoma).
Muhammad Assam et al. (2021) [95]	MRI	MRI brain images classification	Harvard medical school database contained 70 images (25 normal and 45 abnormal, which comprised three different kinds of diseases: brain tumor, acute stroke, and Alzheimer disease).
Antor Hashan et al. (2021) [96]	MRI	Brain tumor	MRI images of the brain were collected from various hospitals and compiled in a Kaggle dataset that contained 400 images (230 brain tumors and 170 normal)
Xiuzhen Cai et al. (2021) [97]	Breast X-ray (mammogram)	Breast cancer	The MIAS mammogram database (http://peipa.esex.ac.uk/info/mias.html) (accessed on 21 June 2022). The dataset contained 322 mammography images that were taken from the UK National Breast Screening Program.
Makineni Kumar et al. (2021) [98]	MRI	Lung cancer disease	MRI lung cancer image dataset, which contained normal and tumor images.
Md Riajuliislam et al. (2021) [99]	Medical data (non-image)	Thyroid disease (hypothyroid)	The dataset from the registered diagnostic center Dhaka, Bangladesh, contained 519 data with 9 attributes.
Aasawari M. Patankar et al.(2021) [100]	Fundus images	Ophthalmic diseases	The dataset was gathered by the authors and contained various ophthalmic diseases such as macular degeneration, retinopathy, myopia, cataract, and other abnormalities.
Deepak R. Parashar et al. (2021) [101]	Retinal fundus images	Glaucoma classification	RIM-ONE dataset contained 455 fundus photographs. Drishti-GS1 dataset contained 101 retinal images. RIM-ONE released 1 dataset which contained 40 images.
Majdah Alshammari et al. (2021) [102]	MRI	Alzheimer’s disease	Alzheimer’s dataset (available: https://www.kaggle.com/tourist55/alzheimers) (accessed on 22 June 2022) contained 4 classes of diseases (896 mild demented, 64 moderate demented, 2240 very mild demented, and 3200 non-demented).
Mohammed Al-Smadi et al. (2021) [103]	Fundus images	Diabetic retinopathy	Kaggle dataset (available: https://www.kaggle.com/c/aptos2019-blindness-detection) (accessed on 22 June 2022) contained 3562 images and was obtained from various clinics in India and represents real-world data.
Saeed Mohagheghi et al. (2021) [104]	X-ray CT	COVID-19 disease	Kaggle dataset contained 1400 healthy and pneumonia images. J. Cohen’s COVID-19 dataset contained 210 COVID-19, normal, and pneumonia images.
Sonit Singh et al. (2021) [105]	CT, MRI, X-ray, PET, US, Microscopy images	Classifying medical image modalities	The authors downloaded 10,000 medical images for each modality from Open-i Biomedical Image Search Engine, National Institute of Health, and U.S. National Laboratory of Medicine.

**Table 2:** Distribution of studies for different feature extraction methods.

Studies (Author (Year) [Ref])	Type of Features	Method Used
Syrine Neffati et al. (2017) [67]	Texture features	DWT transforms to extract features. The kernel PCA (KPCA) technique for feature reduction.
Varun Jain et al. (2017) [68]	Texture features	DWT transform for feature extraction. PCA technique for diminishing the number of features.
AnjKrishna M et al. (2017) [69]	Texture features	SFTA and modified SFTA algorithms to extract features.
Shouvik Chakraborty et al. (2017) [70]	Several key points such as features from which it creates a descriptor for that point	SIFT to detect key (interest) points and feature extraction. Bag-of-features concept to decrease the number of key points.
Priyanka Lodha, et al. (2018) [72]	Cognitive and biological features	The full volume of the brain is extracted from MRI images and other cognitive and biological features.
Keerthana T K et al. (2018) [73]	Texture features	GLCM for features extraction.
Latika A. Thamke et al. (2018) [74]	Texture and shape features Pixel coefficient value	GLCM for features extraction. Moment Invariant (MI). WHT transforms to calculate the pixel value of the image.
Pallavi. B et al. (2019) [76]	Combination of texture and color features	Nine features are mean, standard deviation, entropy, RMS, variance, smoothness, kurtosis, skewness, and inverse difference momentum.
Bahaa Rabi et al. (2019) [78]	Texture and shape features	DWT transforms for feature extraction. HOG transforms for feature extraction. PCA and SVD methods for feature reduction.
Sannasi Chakravarthy et al. (2019) [81]	Texture features	GLCM for feature extraction. CCSA for feature selection manually.
Aamir Bhat et al. (2019) [82]	Texture features	HOG and DWT transform for feature extraction.
Maciej Szymkowsk et al. (2020) [84]	Biometric features	Extract the characteristic points (so-called minutiae) on retina images and count the number of minutiae on the resulting image.
Shashank Awasthi et al. (2020) [85]	Combination of fractal and statistical features	The features such as mean of zero crossing, mean of IMF, standard deviation, etc. PCA for feature reduction.
Halebeedu Suresha et al. (2020) [86]	Texture features	HOG transforms for feature extraction.
Chiranji Lal Chowdhary et al. (2020) [87]	Vital features for segmentation Texture features for classification	The vital features are texture, shape, margin, and intensity. The gray-level histogram computations compute the texture features.
Jaspreet Kaur et al. (2020) [89]	Texture features	GLCM technique for feature extraction.
Nasr Gharaibeh et al. (2021) [92]	Texture and shape features	Haralick and shape-based features. US-PSO-RR algorithm for feature reduction.
Mehedi Masud et al. (2021) [94]	Texture features	2D Fourier Features (2D-FFT) and 2D Wavelet Features (2D-DWT).
Muhammad Assam et al. (2021) [95]	Color features	DWT transforms for feature extraction. Color Moments (CMs) to reduce the number of features.
Xiuzhen Cai et al. (2021) [97]	Texture features	Combination of GLCM and DWT.
Makineni Kumar et al. (2021) [98]	Shape features	Diameter, Perimeter, Entropy, Intensity, and Eccentricity.
Md Riajuliislam et al. (2021) [99]	Data of patients	Age, sex, ID, etc. PCA for feature selection.
Aasawari M. Patankar et al. (2021) [100]	Texture, color, and edges features	Wavelet transform, DCT approach, and color information.
Deepak R. Parashar et al. (2021) [101]	Texture features	Texture-based Zernike moment, chip histogram, and Haralick descriptors.
Sonit Singh el at. (2021) [105]	Statistical and texture features	Local binary pattern (LBP) and GLCM.

**Table 3:** Frequency count of performance metrics utilized in every chosen study.

Studies [Ref]	Performance Metrics	No. of Studies
[68,69,71,73,75,76,77,84,85,89,95,100,102,103,105]	Accuracy	15
[67,79,81,88,92,93,97,101]	Accuracy, Specificity, Sensitivity	8
[70,82,86,94]	Accuracy, Precision, Recall, F1 score	4
[72,74,98,104]	Accuracy, Specificity, Sensitivity, Precision, Recall, F1 score	4
[90,96]	Accuracy, F1 score	2
[66]	Accuracy, Specificity, Sensitivity, AUC	1
[80]	Accuracy, Specificity, Recall	1
[78]	Accuracy, Specificity, Precision, Recall, F1 score	1
[87]	Accuracy, Sensitivity	1
[83]	Accuracy, Specificity	1
[99]	Accuracy, Specificity, Sensitivity, F1 score	1

**Table 4:** Distribution of studies for the most common ML classification methods.

Studies (Author (Year) [Ref])	Techniques	Task	Accuracy Results
Anju krishna M et al. (2017) [69]	Naïve Bayes and SVM	Classify liver tumor: Normal, Cirrhosis, HCC, and Hemangioma	78% 92.5%
Priyanka Lodha, et al. (2018) [72]	SVM, Gradient boosting, NN, K-NN, and RF	Alzheimer’s disease	97.56% 97.25% 98.36% 95% 97.86%
Keerthana T K et al. (2018) [73]	SVM	Diagnosis and classification of brain tumor disease	The system provided better accuracy with the genetic algorithm GA-SVM.
Latika A. Thamke et al. (2018) [74]	K-NN, Multiclass-SVM, and DT	Classify lung disease kinds: Normal, Bronchitis, Pleural, Emphysema, and Effusion	The K-NN classifier gave better outcomes (97.5%) than other classifiers.
Abid Sarwar et al. (2018) [75]	ANN, SVM, K-NN, Naïve Bayes, and Ensemble	Diabetes type-II	The results showed that the ensemble technique provided a superior accuracy of 98.60%.
Pallavi. B et al. (2019) [76]	Multi-level SVM	Classify skin disease	A combination of texture and color features outcomes in the highest classification accuracy using multi SVM.
Neeraj Kumar et al. (2019) [77]	SVM and NN	Bone disease prediction of osteoporosis	Both classifiers gave efficient outcomes.
Bahaa Rabi et al. (2019) [78]	SVM, K-NN, LD, and DT	Classify eight GI classes	The highest accuracy of classification was 99.8%, using the decision tree.
Sannasi Chakravarthy et al. (2019) [81]	PNN	Lung cancer at the early stage	90%
Aamir Bhat et al. (2019) [82]	SVM and ANN	Knee osteoarthritis in early stage	85.33% 73.82%
Maciej Szymkowski et al. (2020) [84]	SVM (linear), SVM (3rd-degree polynomial), K-NN, and K-Means	The healthy and unhealthy	95.25% 96.45% 73.96% 80.42%
Shashank Awasthi et al. (2020) [85]	Logistic regression, Naïve Bayes, and SVM	Alzheimer’s disease classification in MRI image	81% 79.88% 92.34%
Chiranji Lal Chowdhary et al. (2020) [87]	DT, RSDA, SVM, and FSVM	Detecting breast cancer	82.5% 96.1% 88.13% 98.85%
Jaspreet Kaur et al. (2020) [89]	SVM and K-NN	Detecting cancer (cancerous and non-cancerous)	The accuracy of SVM varied from 95.5–98%. Accuracy of t h e K- NN classifier varied from 69.5–95.5%.
Begüm Erkal et al. (2020) [90]	Random Forest, K-NN, Bayes, LMT, DT, and Multilayer Perceptron.	Detecting brain cancer	The experimental outcomes suggest that the Multilayer Perceptron approach outperforms other machine learning methods in accuracy.
Md Riajuliislam et al. (2021) [99]	SVM, DT, RF, Naïve Bayes, and Logistic egression	Hypothyroid at the early stage	99.35% 99.35% 99.35% 94.23% 99.35%
Deepak R. Parashar et al. (2021) [101]	Multi-stage classifier (SVM)	Glaucoma classification	91%

**Table 5:** Summary of articles that included deep learning in detecting diseases.

Studies (Author (Year) [Ref])	Architecture	Task	Results
Danni Cheng et al. (2017) [66]	CNN + RNN	Classify AD vs. NC Classify MCI vs. NC	91.19% (Accuracy), 91.40% (Sensitivity), 91.00% (Specificity), and 95.28% (AUC). 78.86% (Accuracy), 78.08% (Sensitivity), 80.00% (Specificity), and 83.90% (AUC).
Smir S. Yadav et al. (2019) [80]	VGG16 InceptionV3 Capsule Net	Classify pneumonia vs. normal	0.923 (Accuracy), 0.926 (Specificity), and 0.923 (Recall) 0.824 (Accuracy), 0.846 (Specificity), and 0.824 (Recall)
Mohammed Aledhari et al. (2019) [83]	VGG16 ResNet-50 Inception v3 (fine-tuned VGG16)	Pneumonia vs. normal	68% (Accuracy) 58% (Accuracy) 53% (Accuracy) 75% (Accuracy)
Maciej Szymkowski et al. (2020) [84]	ResNet50	Retina diagnosis	86% (Accuracy)
Halebeedu Subbaraya et al. (2020) [86]	CNN + Adam Optimizer	Classify brain tumors	90% (Accuracy) and 0.89 (F1 score)
Laiba Zubair et al. (2021) [93]	CNN+ Bayesian optimization	AD vs. CN vs. MCI	99.3% (Accuracy)
Mehedi Masud et al. (2021) [94]	CNN	Five types of lungs and colon cancers	96.33% (Accuracy), 96.39% (Precision), 96.37% (Recall), 96.38% (F-Measure)
Xiuzhen Cai et al. (2021) [97]	CNN + Thermal Exchange Optimizer	Breast cancer diagnosis	93.79 (Accuracy), 96.89 (Sensitivity), and 67.7 (Specificity).
Makineni Kumar et al. (2021) [98]	LTD-CNN	Lung tumor detection	96% (Accuracy), 96% (Sensitivity), 93% (Specificity), and 94% (Precision).
Majdah Alshammari et al. (2021) [102]	CNN + ML+ Adam Optimizer	AD vs. mild demented vs. moderate demented vs. very mild demented, vs. non-demented	98% accuracy for testing and 97% in training.
Mohammed Al-Smadi et al. (2021) [103]	ResNet, Inception V3 Inception V4 DenseNet Xception EfficientNet	Diabetic retinopathy diagnosis	77.6% (QWK) 82% (QWK) 79.6% (QWK) 81.8% (QWK) 80.9% (QWK) 80% (QWK)
Saeed Mohagheghi et al. (2021) [104]	CNN with CBMIR	COVID-19 disease diagnosis	97% (Accuracy), 99% (Sensitivity), 99% (Specificity 97% (Precision), 99% (Recall), and 98% (F-Measure).
Sonit Singh el at. (2021) [105]	VGG-16 VGG-19 ResNet-50 Inception-v3 Xception MobileNet Inception-ResNet v2	Classifying medical image modalities	62% (Accuracy) 98.18% (Accuracy) 90% (Accuracy) 99% (Accuracy) 98.36% (Accuracy) 98.73% (Accuracy) 98.18% (Accuracy)

## Data Availability

The study does not report any data.

## Abbreviations

CT—Computed Tomography; MRI—Magnetic Resonance Imaging; US—Ultrasound; PET–Positron Emission Tomography; GI—Gastrointestinal Diseases; FCM—Fuzzy C-Mean; IFCM—Intuitionist Fuzzy C-Mean; PFCM—Possibilistic Fuzzy C-Mean; IPFCM—Intuitionist Possibilistic Fuzzy C-Mean; MSW-FCM—Modified Spatial Weighted Fuzzy C-mean; DWT—Discrete Wavelet Transform; PCA—Principal Component Analysis; KPCA—Kernel Principal Component Analysis; SFTA—Segmentation-based Fractal Textural Analysis; SIFT—Scale Invariant Feature Transform; GLCM—Gray Level Co-occurrence Matrix MI- Moment Invariant; WHT—Walsh Hadamard Transform; HOG—Histogram of Oriented Gradients; SVD—Single Value Decomposition; CCSA—Chaotic Crow Search Algorithm; 2D-FFT—Two Dimension Fourier Features Transform; 2D-DWT—Two Dimension Wavelet Features Transform CM—Color Moment; SVM—Support Vector Machine; RBFNN—Radial Basis Function Neural Network; ANN—Artificial Neural Network; SVM-FA—Support Vector Machine with Firefly Technique; DNN—Deep Neural Network; MLC—Maximum Likelihood Classifier; FF-ANN—Feed-Forward Artificial Neural Network; K-NN—K Nearest Neighbor; LDA—Linear Discriminate Analysis; PNN—Probabilistic Neural Network; RSDA—Rough Set DATA Analysis; FSVM—Fuzzy Support Vector Machine; CNN—Convolutional Neural Network; RF—Random Forest; DT—Decision Trees; ROI—Region Of Interest; RNN—Recurrent Neural Network.
